# Psychological well-being of students living in russia and azerbaijan, depending on their native language

**DOI:** 10.1192/j.eurpsy.2021.1249

**Published:** 2021-08-13

**Authors:** Y. Zinchenko, L. Shaigerova, A. Dolgikh, O. Almazova, R. Shilko

**Affiliations:** Faculty Of Psychology, Lomonosov Moscow State University, Moscow, Russian Federation

**Keywords:** native language, mental well-being, multilingualism

## Abstract

**Introduction:**

Since the post-Soviet space is distinguished by a wide ethnolinguistic diversity with Russian language being the basis of identity for a significant part of the population, the role of ethnolinguistic identity in psychological well-being needs deep analysis.

**Objectives:**

The study explores the relationship between mental well-being and belonging to different ethnolinguistic categories in Russia and Azerbaijan.

**Methods:**

The Warwick-Edinburgh Mental Well-Being Scale (Tennant et al., 2007) was used as a measuring tool. The study involved 90 students, 45 participants from multilingual regions of Russia and 45 Russian-speaking students from Azerbaijan. Both samples included three categories of subjects: indicating Russian (1); one or more regional languages in the Russian sample or Azerbaijani in the sample from Baku (2); two native languages - Russian and one of regional languages or Russian and Azerbaijani (3) - as their native language.

**Results:**

No significant differences were observed in the level of psychological well-being in both Russian (KW = 0.594; p = 0.743) and Azerbaijanian students (KW = 1.535; p = 0.464). However, the level of psychological well-being in Russian students from multilingual regions, who indicate the regional language as their native language, is significantly higher than in Azerbaijani students, whose native language is Russian (U = 55,000; p = 0.045).

**Conclusions:**

The sociocultural context is reflected in mental well-being of the individual, depending on his native language and ethnocultural identity. The reported study was funded by RFBR, project number 17-29-09167.

